# Giving Voice to Women with Substance Use Disorder: Findings from Expressive Writing About Trauma

**DOI:** 10.1089/whr.2023.0173

**Published:** 2024-03-12

**Authors:** Nancy Jallo, Patricia A. Kinser, Michelle Eglovitch, Nicola Worcman, Parker Webster, Anika Alvanzo, Dace Svikis, Sarah Meshberg-Cohen

**Affiliations:** ^1^ Department of Family and Community Health Nursing, Virginia Commonwealth University, Richmond, Virginia, USA.; ^2^ Department of Psychology, Virginia Commonwealth University, Richmond, Virginia, USA.; ^3^ Interdisciplinary Cooperation for Ayahuasca Research and Outreach, State University of Campinas, Campinas, Brazil.; ^4^ Chinle Comprehensive Healthcare Facility, Indian Health Service, Chinle, Arizona, USA; ^5^ Substance Use Disorders Consultation Services, School of Medicine, Johns Hopkins University, Baltimore, Maryland, USA.; ^6^ Department of Psychiatry, Yale University School of Medicine, West Haven, Connecticut, USA.

**Keywords:** trauma, substance use disorder, expressive writing, addiction, comorbidity

## Abstract

**Background:**

Trauma exposure is a risk factor for substance use disorders (SUD) among women. This study explores written content from an expressive writing (EW) intervention conducted within a residential SUD program to examine themes across trauma experiences and characterize their deep insight into such experiences.

**Materials and Methods:**

This qualitative study is a secondary data analysis of written content of the first writing session from women (*n* = 44) randomized to an EW condition while in residential SUD treatment.

**Results:**

Nearly all participants (72.7% African American; mean age 37.3 years) reported a significant trauma event (93.2%) with an average of 3.7 types of trauma events (54.4% had a current posttraumatic stress disorder diagnosis). Four primary themes emerged: (1) trauma across the lifespan; (2) loss of safety; (3) altered self-concept; and (4) desire to move on. Most participants identified interpersonal trauma, especially at an early age, as well as parental neglect and physical and/or sexual violence. *These themes indicate a pattern of interpersonal betrayal and paint a picture of trauma and the subsequent “rippling effect” such that the physical, mental, and emotional consequences were often as impactful as the event itself. However, there was also a desire to move on and gain a sense of normalcy.*

**Conclusions:**

Findings highlight the importance of the written word and addressing underlying trauma in addiction treatment to facilitate healing and the woman's desire to move on.

## Introduction

In the United States, substance use disorder (SUD) is a major public health crisis taking a toll on the individual, family, and community.^1^ Affecting over 20 million Americans, SUD is associated with a number of major medical conditions leading to increased risk for reduced quality of life, physical and emotional health comorbidities, and early mortality.^2–5^ In addition, SUDs are among the costliest public health problems, with an estimated loss of over $420 billion a year in the form of health care, lost economic productivity, and criminal justice system costs.^6,7^

Women are the fastest-growing group of substance users in the United States.^8^ This increase in prevalence of women with SUDs has unique serious adverse health consequences given that such consequences are particularly ominous for women. Compared to men, the prevalence of fatal overdoses in women has increased at a higher rate.^9–11^ Notably, women face distinctive issues using substances to deal with emotional and physical pain as well as attempt to self-treat mental health problems, often as a result of experiencing trauma.^11^

There is a heightened vulnerability among women who, compared to men, experience higher rates of lifetime trauma, including physical and sexual abuse.^12–14^ While childhood abuse is more likely among men with SUD, women may be more likely to be exposed to trauma at any age, and trauma is particularly a risk factor for development and severity of SUD among women.^15,16^ For example, traumas such as child abuse have been reported to be associated with earlier initiation and escalation in substance use in women.^17,18^

Traumatic events can lead to prolonged psychological distress and posttraumatic stress disorder (PTSD).^19,20^ Such distress may produce a cycle of avoidance behaviors.^21^ Expressive writing (EW) is an exposure-based approach that targets such avoidance by directly exploring an event and the resultant emotions and thoughts. Typically, individuals are asked to write about their traumatic experiences and their feelings for 15–20 minutes at a time over the course of several days.^19^

According to Pennebaker, who first investigated therapeutic journaling for traumatic events, what makes writing therapeutic is that the writer openly acknowledges their emotions and becomes able to give voice to blocked feelings and to construct a meaningful story in a safe environment. Evidence indicates that disclosure of traumatic and emotional experiences through EW has physical and psychological benefits, and the narratives offer insight for the participant as well as the health care provider (9–11). Written self-disclosure enables people to transfer thoughts to paper, which facilitates meaning making and soothe negative emotions.^22^

Because EW is a type of storytelling in which sensations, thoughts, and emotions are prioritized, it emphasizes emotional truth over objective truth.^23^ Mechanistically, researchers have noted that it is the reflective component in written meaning-making that contributes to changes in narratives and development of adaptive emotions.^24^ In EW, these completely self-driven narratives can achieve a power to garner understandings and shape self-identity (*e.g.*, Syed and McLean, 2015).^25^ Thus, it is not the accuracy of memory so much as the selection and connection of lived experiences that shape the sense of self.

Thus, the content of EW output can provide valuable insight, particularly into women with SUD. Treatment-seeking women with SUD who report trauma histories also report high rates of depression,^26,27^ guilt and blame,^28,29^ disruption of intergenerational family dynamics,^26^ and transgenerational patterns of trauma.^30^ This study provided a unique opportunity to explore these factors through the women's voices and analyze EW content that was not written with the intention of providing researchers with qualitative data; rather, was intended to help facilitate processing.

### Purpose

The purpose of this qualitative study was to explore the written self-reported experiences and perceptions of trauma among women in residential SUD treatment facing the challenges of addiction. Our aim was to give voice to these women and characterize their deep insight into lived experiences.

## Materials and Methods

### Research design

This study is a secondary data analysis focused on content from the first writing session of women randomized to EW while in a residential SUD treatment facility. The primary outcome data have been previously reported.^31^ Virginia Commonwealth University (VCU)'s Institutional Review Board approved the research.

### Procedure

Participants (*N* = 149) were recruited during the first several days of residential treatment. Participants were randomly assigned to the expressive or control writing condition. EW instructions, adapted from Pennebaker's paradigm,^32^ instructed participants to write for 20 minutes about *your deepest emotions and thoughts about the most traumatic experience in your life. In your writing, I'd like you to really let go and explore your very deepest emotion and thoughts. You might tie this trauma to your childhood, your relationship with others including your parents, lovers, friends, or relatives. You may also link this event to your past, present, or your future, or to who you have been, who you would like to be, or who you are now.*^31,33^ Writing instructions were read aloud to each participant before every session and a copy of the writing instructions was provided. Following 20 minutes of writing, participants placed their journals, identified by participant ID, into a cardboard box.^31^ The data were collected from June 17, 2007, through November 8, 2008.

### Data analysis

This study uses qualitative descriptive approach using conventional content analysis to examine transcribed written data for *n* = 44 women assigned to the EW condition. Qualitative description is data-derived, to conceptualize participants' experiences using their own words^34–36^; we used an inductive conventional content analysis approach.^34^ This approach is intended to let the written voices speak for themselves, rather than depending upon *a priori* coding or deep interpretation of the researcher.

Four research team members engaged in analysis. Driven by the stepwise approach described in Coloarafi and Evans (2016),^34^ the team engaged in the following process: First, analysts read transcripts independently in their entirety to garner a sense of the data. Second, analysts read transcripts line by line and assigned codes to key concepts. Third, the team met to review the coding, which was combined and grouped into key themes. Fourth, team members conducted another analysis of the data independently, by re-reading the entire transcripts and ensuring that the coding and key themes accurately represented the data. Finally, the team connected relationships between themes and identified quotes. To ensure scientific rigor, the team comprehensively read and analyzed the data, employed team-based decision-making about coding and themes, and created an audit trail during all meetings.^37,38^

## Results

### Sample description

Table 1 summarizes the participants' sociodemographic characteristics. The mean age of participants was 37.3 years (standard deviation [SD] = 8.8), and the majority identified their race as African American (72.7%). The average years of education was 11.2 years (SD = 1.4), and the majority were single/never married (65.9%). Many met *Diagnostic and Statistical Manual of Mental Disorders* 4th edition *(DSM-IV)* SUD criteria for more than one substance (68.2%), with 81.1% reporting a cocaine use disorder. The next most commonly diagnosed SUD was opioid (43.2%), followed by alcohol (38.6%). Overall, 54.4% met criteria for current PTSD *via* the Posttraumatic Diagnostic Scale^39^; with an average of 3.7 (SD = 2.3) different types of trauma events, with nearly all participants (93.2%) reporting at least one trauma event significant enough to meet PTSD. Four participants explicitly linked addiction to their traumatic experiences.

**Table 1.** Participant Characteristics

Characteristic	*Percent or M (SD) *N* = 44*
Age (years)	37.25 (8.8)
Education (last grade completed)	11.2 (1.4)
Marital status, *n* (%)	
Married	13.6
Single/never married	65.9
Divorced/separated	20.5
Race, *n* (%)	
Caucasian	22.7
African-American	72.7
Hispanic	0.0
Other	4.5
Employment status, *n* (%)	
Unemployed	81.1
Full-time work	13.6
Part-time work	4.5
Number of different types of trauma events	3.7 (SD = 2.3)
DSM-IV substance dependence diagnosis, *n* (%)	
Alcohol	38.6
Cannabis	9.1
Cocaine	81.8
Hallucinogen	2.3
Opioid	43.2
More than one drug	68.2

DSM-IV, *Diagnostic and Statistical Manual of Mental Disorders* 4th edition; SD, standard deviation.

### Emergent themes

Four primary themes emerged, including (1) trauma across the lifespan, (2) loss of safety, (3) altered self-concept, and (4) desire to move on.

#### Theme 1: Trauma across the lifespan

Common traumas included physical/sexual violence, either experienced or witnessed, and psychological abuse. Overall, 42 (95.5%) women described at least one of these traumatic experiences. Two women who did not describe violence or abuse depicted experiences such as difficulties with parental and social relationships.

While trauma may have occurred throughout their lives, most women described key events occurring during childhood. Physical abuse was often by mothers or fathers and included being beaten and burned, as illustrated by comments, such as, “*my mom would beat me with a belt,*” “*my mom burnt me when I was 2 years old and use to beat us*,” “*my father was an alcoholic and would beat us and have sex with his own girls.*” In addition, participants were often threatened with harm to them or a family member if they told anyone what they witnessed, resulting in multiple forms of abuse.

Women also described sexual abuse involving a high level of familiarity between them and the perpetrator. Often, the perpetrator was a family member or close friend. One participant wrote, *The most traumatic experience in my life was when I was four years old. At that age my brother, who was twelve, sexually assaulted me*. Another wrote, *I was about 6 [years old], and my stepdad came into my room to put me to bed and touched my private areas … he continued to molest me until I was about 10–12.* Another participant was raped at 18 years of age by her boyfriend's friend, and another was raped at 13 years of age by her mother's boyfriend's friend.

As adults, participants experienced physical abuse, with one woman describing she *married at 15 to get away from abusive home* and soon realized he *turned out to be an alcoholic and wife beater*. Witnessing violence and the aftermath was also experienced as an adult. As one participant described her most traumatic experience *my friend got shot when I was standing there. I feared for my life and my children's life.* Likewise, another participant witnessed her parents murdered in their home and *seeing my dad laying [in] the bed in a pool of blood and my mother was trying to fight …. I remember seeing my mother get stabbed to death.*

#### Theme 2: Loss of safety

Loss of emotional and/or physical safety was expressed as a form of betrayal in many of the written entries. Many women wrote about traumatic scenarios within a backdrop of an unsafe physical environment and a lack of protection, especially as children. Those who experienced child abuse remember a lack of support or protection by family members during or after these experiences. One woman wrote about being sexually assaulted as a child by her stepfather and wondered why her mother did not protect her: *I wondered where my mom was, and did she know what was really going on.* This lack of protection became apparent when she was older and realized her mother knew what was happening—her mother told her to *allow him to play—touch me.*

Similarly, one woman described a sense of betrayal when her parents allowed her grandfather to move in even though they knew he had a history of assaulting children. *As a child between the age of 8–11, I was sexually molested by my grandfather who was living in our house—My mother years later informed me that my grandfather had been accused of molesting other children and he had to move in so he wouldn't be arrested …. my parents never warned me or my brother. I never felt so betrayed.*

Participants verbalized that lack of safety also occurred often in the form of parental neglect. When, as children, they were left unattended at home, participants reported being sexually assaulted by friends: *My mother was at work, I was at home by myself scared to death*. As consequences of abuse and neglect, participants wrote about being *taken away* and *entered into foster care* or *adopted to another family.* Pervasive drug use in their family, friends, or community added to loss of safety. Writings included, *my brother was selling drugs out of the house, and it wasn't really a safe environment for no one*.

#### Theme 3: Altered self-concept

Consequences of the trauma experiences included a range of emotions, best described as altered self-concept. Self-concept is the image people have of themselves and is influenced by many forces, including trauma.^40^ Trauma can profoundly affect the sense of self, where both cognitive and somatic disturbances to the sense of self are reported clinically by individuals.^40^ Overall, many participants described the emotional impact of exposure to both psychological and/or physical violence. They often voiced how their identity was shaped by these experiences, as one participant explained: *I feel that self-image is greatly affected by the relationships we have.*

Emotional changes were expressed in different ways. Many participants expressed a generalized feeling of being inferior due to repetitive loss of safety over time. The sense of loss of self-esteem was evident as women expressed a general sense of deserving their current situation and/or the traumatic events, often playing out in dysfunctional relationships with others. Participants reported crossing their limits in unhealthy relationships, as exemplified by *I cannot have normal relationships because of my own self- which is very poor, no matter what I do in my life.*

Relatedly, many women shared a feeling of not being loved or wanted by others. For example, one participant revealed that being a victim of physical and psychological abuse from her father resulted in not feeling self-love: *I felt loss and unloved because of this, I never had a childhood and still feel I missed a lot.* Parental abandonment may impact the construction of the self because it produces a ripple effect on the children. First, the feeling of a lack of protection leading to doubt: *Wasn't I loved?* Next, the feeling of self-blame contributing to this abandonment, as one explained: *One of the most traumatic experiences I have had in my life is the fact that my birth mother gave me up for adoption. I never understood the reason why. I have asked my birth mother time after time, but she would never give me an answer. I wonder, was it something that I did?*

Participants often described anger as a result of such experiences, ranging from hatred of a specific person that played a central role, as described by a woman who witnessed her father's violent behaviors toward her sister and mother: *I felt helpless afraid and that turned into hate, hate for my dad. I hated my dad for many reasons. How could someone inflict so much pain to an entire family?*; to anger targeting themselves, as illustrated by another participant after being raped by her boyfriend: *I felt in anger, and I directed it towards myself as if it was my fault.* Guilt was also commonly expressed, and some commented that they blamed themselves, including one participant, who reported that after witnessing her parent's murder: *I blamed myself for a long time. I felt like I should've been the one dead, not my parents.*

#### Theme 4: Desire to move on

Despite the trauma these women faced, a common theme was the desire to move on, regain a sense of normalcy, and look toward the future. For example, one participant commented, *[the trauma] keeps me sick because I hold on to it instead of working through it. I don't want to carry this weight anymore. I need help and am willing to do whatever it takes to get better … I am sick and tired of being sick and tired.*

Women also wrote about efforts to establish positive relationships with close family members by whom they felt betrayed. One woman who identified being sexually assaulted as a child by her older brother stated, *Now I am learning to trust him more so we can be a family again. In the future, I want us to be like one with family and watching our nieces and nephews grow up, and rock rocking chairs on the front porch of my house.* Another participant, after detailing a rape by her two older brothers and the loss of her family wrote, *I don't know my real family—I want to get to know them again—I want my family back.*

Lastly, participants identified a desire to forgive themselves to move on. One participant who did not report a sexual assault to the police wrote, *I want to forgive myself for all the women he raped after me because I was scared of people knowing and not being popular or believed.* Another stated, *I wish it hadn't happened to me, but it did, so now I have to deal with it and go on with my life and learn that it wasn't my fault and tell myself that I didn't deserve what happened.*

## Discussion

This study examined written content from the first writing session in a sample of women who engaged in a study examining EW as a therapeutic process among women in residential SUD treatment. While not seeking treatment for PTSD or trauma, results demonstrated that most women clearly experienced significant trauma events with far-reaching effects. One of the main findings of this qualitative study was the “ripple effect” of trauma. In these women, trauma experience appeared to ripple over the life-course, negatively impacting participants' physical, mental, and emotional well-being. This image provides a lens through which to see the challenges of trauma and substance use in these women and provides a possible direction for treatment and intervention development.

Consistent with previous research, these participants identified multiple forms of emotional and physical interpersonal trauma (IPT) experiences throughout their life.^3,28,29,41–44^ This is noteworthy, as IPT is considered among the most detrimental forms of trauma and is associated with increased somatic symptom severity, SUD, suicide risk, and overdose.^45–49^ IPT victims are at higher risk for experiencing cumulative trauma and re-victimization, increasing the probability of additional substance issues.^45,50,51^

Adverse childhood experiences (ACE), a form of IPT, can have long-lasting negative effects on psychosocial development including SUD.^52^ Our results are consistent with previous research revealing significant rates of ACEs among women with SUDs.^3,30,53–55^ ACEs, such as physical abuse, emotional and physical neglect, parental substance abuse, parental separation, and death, were voiced by participants in this study. Findings emphasize the extensive nature of childhood abuse and neglect and may underscore a self-medication hypothesis to suggest why some women turn to drugs and alcohol to cope with consequences from childhood abuse and stress feelings related to exposure of ACEs.^3,56^

Another common theme in this study was the desire to move on, which underlined complicated relationships with forgiveness of perpetrators of such events. These findings are consistent with literature suggesting that the concept of forgiveness in the face of abuse is highly complex. Difficult to define, forgiveness may involve compassion, reconciliation, and/or a reframing of the offense from negative to either positive or neutral.^57^ Researchers found that forgiveness was associated with psychological health in women with history of abuse, but not in women currently experiencing abuse; in fact, forgiveness was associated with increased stress when physical abuse was ongoing.^58^ Future research is warranted to explore the concept of forgiveness with women with SUD who have experienced IPTs.

Notably, despite being in SUD treatment, only four participants explicitly discussed an association between their traumatic experiences and their addiction in this first writing session. Use of substances as a maladaptive self-medication strategy is well supported in the literature.^59,60^ Thus, these lack of findings may reflect a limitation in the study design as the data are derived from written journal entries and, thus, the team was unable to ask clarifying questions or elaboration on whether the experience of traumatic events was directly connected to their use of substances. More research is needed in this area to further explicate this finding.

Overall, the emotional resonance of the content of the sessions demonstrates the power of EW and writing for yourself. The advantages of EW can be understood in relation to cognitive, physical, and psychological health.^61^ Guo posits three mechanisms for which this manifests: Cognitively, translating thoughts into writing enables people to symbolize the experience, which enhances processing and understanding.^23^ Physiologically, written disclosure decreases the work of repressing emotions which is associated with stress. Psychologically, confronting the crisis through written word allows individuals to reframe and integrate it into their schema, such that negative emotionality eventually diminishes. This trifold of impact has particular salience with women with SUD, who oftentimes experience multiple traumas.

Thus, this study provides support for future interventions that utilize EW in this population. Written exposure therapy (WET) is a newer form of exposure therapy that builds upon Pennebaker's initial EW paradigm that has been studied primarily in veterans with PTSD.^62^ Recently, Nillni et al. have piloted WET among women with comorbid PTSD and SUD and found that it was effective in reducing PTSD symptoms.^63^ However, there is limited research around how WET might improve substance use outcomes in this population. Future research should continue to explore its effectiveness for this population given the high comorbidity of PTSD and SUD and explore the impact on substance use in addition to PTSD symptomatology.

### Limitations

There are several limitations to this study. Only women with SUD in residential treatment were included and thus are not representative of all women seeking SUD services, limiting generalizability. This study examined the first writing session and further research should investigate the EW entries over time to examine whether there were changes in the way that the women reflected upon written events. Despite this, the present study provides the context and meaning of these traumatic experiences within this subgroup, which is the goal of qualitative research.

## Conclusion

Our findings revealed a complex nature of trauma among women in residential SUD treatment settings. Such findings might highlight the importance of addressing underlying trauma during early stages of SUD treatment. Incorporating a model of informed care that acknowledges the role trauma may play in an individual's life could help contextualize the individual's experience and strengths to tailor behavioral and treatment strategies to better cope with addiction and facilitate healing.^26,64^

## Abbreviations Used

ACE: adverse childhood experiences
EW: expressive writing
IPT: interpersonal trauma
PTSD: posttraumatic stress disorder
SD: standard deviation
SUD: substance use disorders
VCU: Virginia Commonwealth University
WET: written exposure therapy
